# Application of the comprehensive set of heterozygous yeast deletion mutants to elucidate the molecular basis of cellular chromium toxicity

**DOI:** 10.1186/gb-2007-8-12-r268

**Published:** 2007-12-18

**Authors:** Sara Holland, Emma Lodwig, Theodora Sideri, Tom Reader, Ian Clarke, Konstantinos Gkargkas, David C Hoyle, Daniela Delneri, Stephen G Oliver, Simon V Avery

**Affiliations:** 1 School of Biology, Institute of Genetics, The University of Nottingham, University Park, Nottingham NG7 2RD, UK; 2 North West Institute for Bio-Health Informatics, The University of Manchester, ISBE, School of Medicine, Oxford Road, Manchester M13 9PT, UK; 3 Department of Biochemistry, University of Cambridge, Sanger Building, Tennis Court Road, Cambridge CB2 1GA, UK; 4 Faculty of Life Sciences, The University of Manchester, Oxford Road, Manchester M13 9PT, UK

## Abstract

**Background:**

The serious biological consequences of metal toxicity are well documented, but the key modes of action of most metals are unknown. To help unravel molecular mechanisms underlying the action of chromium, a metal of major toxicological importance, we grew over 6,000 heterozygous yeast mutants in competition in the presence of chromium. Microarray-based screens of these heterozygotes are truly genome-wide as they include both essential and non-essential genes.

**Results:**

The screening data indicated that proteasomal (protein degradation) activity is crucial for cellular chromium (Cr) resistance. Further investigations showed that Cr causes the accumulation of insoluble and toxic protein aggregates, which predominantly arise from proteins synthesised during Cr exposure. A protein-synthesis defect provoked by Cr was identified as mRNA mistranslation, which was oxygen-dependent. Moreover, Cr exhibited synergistic toxicity with a ribosome-targeting drug (paromomycin) that is known to act via mistranslation, while manipulation of translational accuracy modulated Cr toxicity.

**Conclusion:**

The datasets from the heterozygote screen represent an important public resource that may be exploited to discover the toxic mechanisms of chromium. That potential was validated here with the demonstration that mRNA mistranslation is a primary cause of cellular Cr toxicity.

## Abstract 2

Competitive growth between over 6,000 heterozygous yeast mutants in the presence of chromium together with microarray-based screens showed that proteasomal activity is crucial for cellular chromium resistance.

## Background

Toxic metals are major environmental pollutants that are linked to a broad range of degenerative conditions in humans [1-3]. Metal toxicity is also widely studied in microorganisms, both as models to further our understanding of cellular metal toxicology, and because of the importance of metal toxicity in microbial biotechnologies [4-7]. Chromium toxicity is an issue of especially broad interest, Cr compounds having been among the earliest chemicals to be classified as carcinogens. Although the consequences of chromium toxicity are well documented [8], the underlying cause(s) of toxicity remains unknown. This is a key issue, as an understanding of mechanism should help develop appropriate therapies.

The yeast *Saccharomyces cerevisiae *is at the forefront of functional genomics and systems biology research [9] and provides an excellent model with which to tackle intractable biological questions. The yeast deletion strain collections have proven particularly valuable resources, the homozygous versions having been used widely for genome-wide assignment of function [10-13]. The heterozygous deletion strain collection [14] has been less commonly exploited. This reflects (in part) the more subtle phenotypes that the reduction in the copy number of a given gene (from two copies to one), as opposed to its complete removal, is expected to produce. This subtlety means that small differences in growth rate of individual heterozygous mutants must be detected, and this is most easily achieved by competition experiments. These experiments are generally carried out by pooling the entire collection of mutants and growing them in competition under the condition of interest [11,15]. Analysis of the competitions is facilitated by the fact that the gene replacement cassette for each mutant has a unique 20-mer strain-identifying sequence [10,14]. These molecular 'barcodes' are amplifiable with common primers, enabling a parallel analysis of all strains in the mixed culture and avoiding the need to culture each strain separately to assess growth effects. Total genomic DNA extracted from the mixed competitions is subjected to PCR with the universal primers, yielding a pool of amplified tag sequences in which the abundance of each unique tag corresponds to the abundance of a strain in the culture [14,16]. These abundances can be determined quantitatively by hybridization to oligonucleotide arrays, the data revealing the relative growth of each yeast mutant under the growth condition(s) of interest.

A major advantage of the heterozygous deletion strain collection is that it encompasses essential gene functions that, by definition, are not represented in the homozygous collections. Therefore, exploitation of the heterozygous mutant collection through competition analyses should provide a considerably richer pool of information. Essential gene products are likely cellular targets of drugs and other xenobiotics. Consequently, existing data from screens of the homozygous mutant collections against agents such as mutagens and toxic metals [17-19], although very useful, exclude potentially key information. Furthermore, mutation to heterozygosity is common in nature, and such heterozygosities can underlie human genetic diseases [20].

The proof-of-principle of competition analyses employing the heterozygous yeast deletion mutants involved confirmation or identification of essential proteins as targets of drug action [14,15,21]. For those purposes, large collections of pooled mutants were co-incubated with the drug and the relative growth effect of the drug on each strain assayed as outlined above. Genes were identified that yielded haploinsufficiency phenotypes. Haploinsufficiency describes the situation where halving the copy number of a gene (to create a heterozygous mutant) provides insufficient gene product for optimal growth under a particular condition. Therefore, the above studies identified (essential) genes that are required for optimal growth in the presence of the drugs, revealing putative drug targets. Another phenomenon, not yet exploited in the above context, is haploproficiency (that is, a fitness benefit arising from heterozygosity). Recent work has highlighted the value of considering haploproficiency. For instance, genes with functions related to protein turnover showed haploproficiency under conditions of nitrogen limitation, where protein conservation might be expected to yield a selective advantage [22].

Armed with these convincing proofs of principle, the present study extends the use of competition analyses, beyond the identification of drug targets, to a natural stressor that is not necessarily expected to have a primary protein target - the toxic metal chromium. Prior to our study intense efforts to characterize the toxic action of chromium have been made, but the primary molecular mechanisms causing toxicity have, nevertheless, remained elusive. Here we show that mRNA mistranslation is a primary cause of cellular Cr toxicity.

## Results

### Identification of heterozygotes with altered chromium resistances

The experimental system involved co-culture of >6,000 heterozygous (hemizygous) deletion strains in carbon-limited continuous culture. A number of similar studies have co-cultured the heterozygotes in batch culture [15,21], whereas a more recent study with the heterozygotes has used the same continuous culture system as that employed here [22]. The use of continuous culture for competitions enables detection of the more subtle phenotypes, expressed as small growth rate differences and revealed over a large number of generations. In addition, use of the chemostat for continuous culture offers high reproducibility, owing to a defined and constant growth rate and physicochemical environment at steady state. CrO_3 _was supplied at a sub-lethal dose (0.1 mM), pre-determined to cause an approximately 30% increase in the mean doubling time of the mixed cultures. The relative growth of each strain in the cultures (that is, change in relative abundance between the start and end of a chemostat experiment) was derived from signals assigned to the strains' unique identifying (barcode) sequences (see Background, and Materials and methods). The effect of Cr was determined by comparing the relative growth of each strain in the Cr-treated cultures versus that in control cultures. This yielded a value for each strain for the size of the growth effect of Cr (see the Data analysis section in Materials and methods). The data for each strain are given in Additional data file 1.

**Figure 1:**
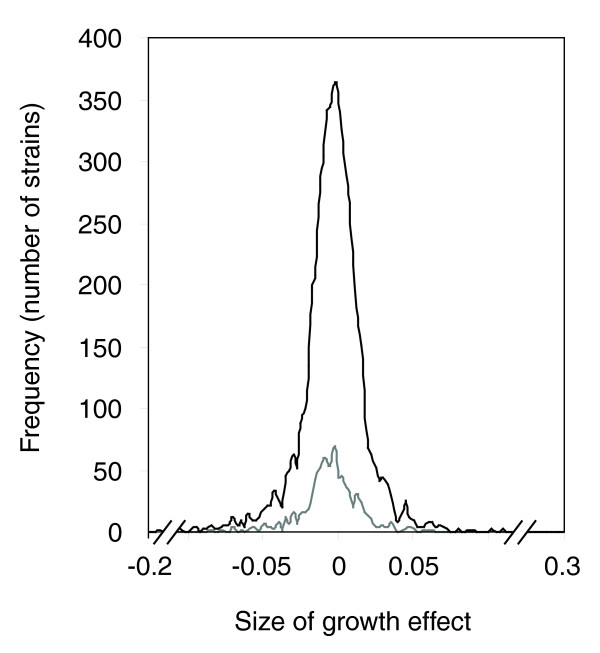
Analysis of the global effects of Cr treatment on the heterozygous mutants. The plot shows the distribution of the sizes of the growth effects caused by Cr for all genes (black line) and essential genes (grey line). Mutants were grouped into bins according to the size of growth effect. Each bin encompasses a 0.0025 range of growth-effect sizes, and the frequency denotes the number of strains in each bin. The calculation for determining size of growth effect is described in the Data analysis section in Materials and methods.

The range of growth effects of Cr across the strains indicated a normal distribution centered around zero (Figure 1). The relative growth of some strains was decreased by Cr (negative growth effect; tendency towards haploinsufficiency with Cr), whereas others showed improved relative growth (tendency towards haploproficiency). A similar normal distribution was evident for growth effects on strains that were heterozygous specifically for essential gene functions. The growth data were analyzed further to identify strains that showed significant (false discovery rate, *q *< 0.05) haploinsufficiency or haploproficiency (see the Data analysis section in Materials and methods). There were fewer significantly haploinsufficient strains than haploproficient ones, that is, 115 strains exhibited a Cr-specific growth defect (indicating gene functions that normally protect against Cr), whereas the relative competitiveness of 203 strains was enhanced by Cr (indicating functions through which metal toxicity could be mediated). This suggests that *S. cerevisiae *has not been routinely exposed to Cr stress during its evolutionary history, as Fisher [23] demonstrated that when selection occurs in the environment to which an organism is adapted, then most mutations will be deleterious; whereas, when selection occurs in increasingly suboptimal conditions, then an increasing proportion of mutations will be beneficial. Knowledge of genes that exhibit haploproficient phenotypes in the presence of Cr could be exploited to increase the rate of biotechnological processes that may be limited by metal toxicity [4,7].

In contrast to the therapeutic compounds that were the subjects of previous haploinsufficiency analyses [14,15,21,24], there is no *a priori *expectation that the primary target of Cr will be a specific essential protein [5]. Nonetheless, closer analysis of our data showed that, under the condition of Cr stress, five of the eight most significant haploinsufficient phenotypes (that is, those with the lowest *q*-values) were found in strains heterozygous for an essential gene (Additional data file 1). These included *NHP2 *(involved in 18S rRNA processing), and *ARP3 *and *ARC19 *(involved in actin nucleation and actin patch function). As with the drugs, these observations could, in principle, be explained by direct interference of the metal with the essential function of its target protein, reducing its activity to a level below that required to sustain the growth of a diploid cell at wild-type rates. Alternatively (and this also applies to the haploinsufficient phenotypes observed for non-essential genes), there may be a synthetic lethal interaction with the principal target of chromium, or the haploinsufficient protein may contribute to the intrinsic resistance of the cell to the toxic action of the metal.

### Over-representation of specific Gene Ontology terms in the annotations of genes found in the haploinsufficiency and haploproficiency datasets

GoMiner [25] was used to associate Gene Ontology (GO) terms with all genes whose heterozygous mutants exhibited significant haploinsufficiency or haploproficiency under Cr stress (Additional data file 2). Not unexpectedly, GO terms related to transport and metal homeostasis were significantly over-represented in the annotation of those genes that displayed either haploinsufficient or haploproficient phenotypes. Gene functions involved with chromatin structure were also significantly over-represented in the haploinsufficient and haploproficient data, indicating an involvement of chromatin organization and its possible effects on gene expression in Cr resistance. Schnekenburger *et al*. [26] have described how Cr cross-links complexes of histone deacetylase 1 and DNA methyltransferase 1 to gene promoters, inhibiting histone modifications and decreasing recruitment of RNA polymerase. Cr may also provoke aberrant DNA methylation, with the potential to silence tumor suppressor genes in higher cells [27]. Genes involved in nucleotide excision repair also were evidently important for Cr resistance, consistent with previous work [28]. Cr is well known to promote DNA damage, but it is unresolved whether this is a primary cause (versus a secondary effect) of Cr toxicity.

The Cr treatment revealed haploproficient phenotypes for several genes involved in sulfur metabolism. The flux of sulfur in these heterozygotes could be re-directed towards molecules that may promote metal resistance, such as glutathione (GSH). Such re-programming of sulfur metabolism occurs normally in wild-type yeast responding to other metals [29,30]. In addition, Cr uptake may occur through sulfate transporters, which are regulated in response to Cr stress [31]. Actin was a highly over-represented haploinsufficient category for Cr, which might relate to targeting of actin function by Cr, as suggested above.

### Proteins synthesized during chromium exposure tend to form aggregated toxic-products

**Figure 2:**
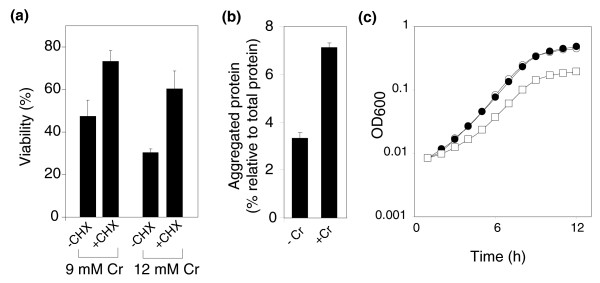
Chromium causes accumulation of toxic protein aggregates. **(a) **Exponential phase cells of *S. cerevisiae *in YEPD medium were exposed to 9 or 12 mM CrO_3 _for 3 h, in either the absence or presence of 10 μg ml^-1 ^cycloheximide (CHX) (the latter cells were also pre-incubated for 1 h with CHX before metal exposure). Viability (%) was subsequently determined according to colony-forming-unit counts, with reference to control cultures not exposed to Cr. **(b) **Protein was extracted from cells treated for 30 minutes with 0.5 mM CrO_3 _and an aggregated protein fraction (separated from soluble and membrane proteins) [49] was prepared from each sample. The data show protein determined in the aggregate fraction as a proportion of the total cellular protein. **(c) **Cells were cultured in YEPD medium that was either unsupplemented with protein (open circles), or supplemented with 24 μg ml^-1 ^of the soluble (filled circles) or aggregated (squares) protein fractions isolated from cells that had been exposed to 0.2 mM CrO_3 _for 1 h. All values are means ± standard error of the mean from at least three independent determinations.

Genes involved in proteasome function and regulation of protein stability were among those most significantly over-represented in the set showing haploinsufficient phenotypes in the presence of Cr (Additional data file 2). We decided to subject this evidence of the mechanism of chromium's toxicity to further investigation. Initially, we validated the output from the library screen by confirming in independent batch-culture assays the haploinsufficient phenotypes of several Cr-treated proteasome mutants (Additional data file 3). These data point to a requirement for protein degradation in Cr resistance and, therefore, to an involvement of cellular proteins in Cr toxicity. This hypothesis was supported by experiments involving cycloheximide, an inhibitor of translational elongation. Exposure of cells to Cr for 3 h resulted in a marked loss of viability (Figure 2a). However, this toxicity was suppressed in cells that were blocked for protein synthesis using cycloheximide.

Previous evidence showed that Cr toxicity involves protein oxidation [32]. Oxidized proteins are prone to forming potentially toxic aggregates [33], but this can be countered by proteasomal degradation of the abberant proteins. Combining those observations with our new haploinsufficiency data, we hypothesized that Cr toxicity could involve the formation of protein aggregates. Protein aggregation is also linked to cancer [33], and the carcinogenicity of Cr is well-documented [8]. To test the effect of Cr on protein aggregation, insoluble aggregate fractions of proteins were isolated from cells (see Materials and methods) that had been incubated with or without Cr, and the levels of protein in these fractions were determined. The proportion of cellular protein occurring as insoluble aggregates was found to increase approximately two-fold during Cr exposure (Figure 2b), indicating that Cr promotes protein aggregation. Protein aggregates can be toxic, and the potential toxicities of aggregate preparations from cells can be tested by exposing fresh cells to these and measuring their inhibitory effect [34] on the growth of *S. cerevisiae*. Growth was not affected by supplementing the medium with soluble protein that was previously isolated from Cr-treated cells (Figure 2c). In contrast, growth was slowed in medium supplemented with an equivalent amount of aggregated protein from the Cr-treated cells. Therefore, aggregated protein formed in the presence of Cr can exert a toxic effect.

**Figure 3:**
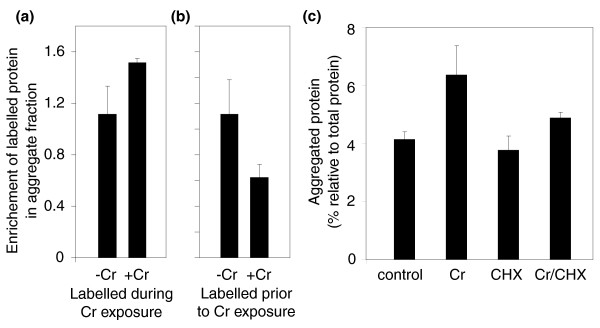
Chromium causes aggregation predominantly of proteins synthesized during chromium exposure. Cells were exposed to 0.1 mM CrO_3 _for 60 minutes, either **(a) **at the same time as or **(b) **after labeling with [^35^S]methionine for 60 minutes. The data show the relative enrichment of isotope in the aggregate fraction [cpm per μg aggregated protein, corrected for labeling efficiency (cpm per μg total protein)]. (b) Due to the natural turnover of labeled proteins during the post-labeling 60 minute incubation ± Cr, the data from this experiment were normalized with respect to the minus-Cr control from (a). **(c) **Aggregated protein as a proportion of total protein was determined after incubation of cells for 1 h in the absence or presence of 0.4 mM CrO_3 _and 10 μg ml^-1 ^cycloheximide (CHX). All values are means ± standard error of the mean from three independent determinations.

The observation that Cr resistance was enhanced by the simultaneous inhibition of protein synthesis (Figure 2a) suggested that proteins synthesized during Cr exposure were involved in toxicity. To explore this further, the source of Cr-induced protein aggregates was determined with pulse-chase experiments involving protein labeling with [^35^S]methionine. These experiments showed that Cr-dependent aggregation was attributable primarily to proteins synthesized during Cr exposure, rather than to aggregation of pre-existing proteins: the aggregate fraction isolated from cells that were [^35^S]methionine-labeled during the period of Cr exposure was enriched with labeled protein (Figure 3a), whereas the opposite was true for cells labeled prior to Cr exposure (Figure 3b). (In the latter case, there was a decrease in the proportion of labeled protein in the aggregate fraction following incubation with Cr. This could be due to dilution of pre-existing labeled aggregates with unlabelled aggregates formed during the incubation with Cr.) In other experiments, co-treatment with cycloheximide suppressed the Cr-dependent accumulation of protein aggregates (Figure 3c), supporting the conclusion that proteins that form insoluble aggregates in response to Cr are predominantly synthesized during Cr exposure.

### Mistranslation of mRNA is a primary cause of chromium toxicity

**Figure 4:**
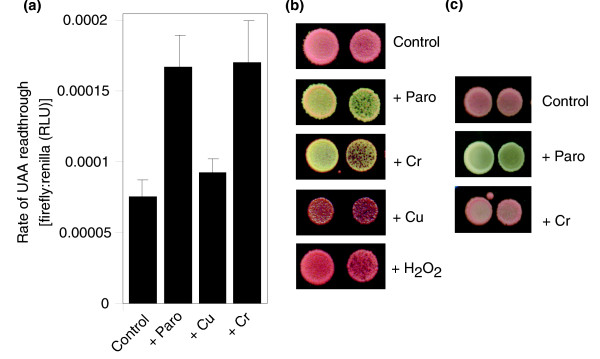
Chromium causes errors in mRNA translation. **(a) **Cells transformed with the dual-luciferase plasmid [50] were exposed or not to 200 μg ml^-1 ^paromomycin ('Paro'), 0.6 mM Cu(NO_3_)_2 _or 0.1 mM CrO_3_, in YNB medium for 90 minutes. The activities of the firefly and renilla luciferases in derived protein extracts were determined luminometrically. The ratio of luminescence from the firefly versus renilla luciferase indicates the short-term level of translational read-through of the UAA stop codon that separates the two open reading frames. All values are means ± standard error of the mean from at least three independent determinations. RLU, relative light units. **(b, c) **Exponential-phase *S. cerevisiae *L1494 (*ade1-14*) cells (OD_600 _~1.0, plus a 10-fold dilution) were spotted in 6 μl aliquots on to YEPD agar supplemented or not with 150 μg ml^-1 ^paromomycin, 8 mM Cu(NO_3_)_2_, 0.15 mM CrO_3 _or 3.6 mM H_2_O_2_. Plates were incubated for 3 days at 30°C either aerobically (b) or anaerobically (c). In the latter case, plates were incubated aerobically at 4°C after the 3 days incubation to allow development of the red pigment before images were captured. The stressors were supplied at doses that produced similar degrees of mild inhibition of aerobic growth (versus controls) within each experiment on the different media.

The finding that Cr causes aggregation primarily among proteins being synthesized during exposure suggested that the metal might be targeting the protein synthesis or folding machineries. Mistranslation of mRNA transcripts provides a major potential source of aberrant proteins that form aggregates [33]. To test whether Cr provokes mRNA mistranslation, the rate of translational read-through of a UAA nonsense (stop) codon was monitored in a short-term dual-luciferase assay (see Materials and methods). The rate of read-through was increased more than two-fold by the addition of CrO_3_, at a concentration that increased the population doubling time by about 15% (Figure 4a). A similarly inhibitory dose of another metal, Cu(NO_3_)_2_, did not significantly affect read-through across the stop codon. The ribosome-targeting drug paromomycin caused a stimulation of mistranslation comparable to that observed with Cr. These data were supported by results from a longer-term qualitative assay, based on read-through of the *ade1-14 *UGA codon and suppression of the red pigmentation associated with this allele. Treatments with agents such as H_2_O_2 _or Cu(NO_3_)_2 _gave no change in colony color compared with untreated controls, whereas red pigmentation was suppressed with paromomycin or CrO_3 _(Figure 4b), indicative of mistranslation [35]. Red pigmentation was restored when pale colonies from Cr-supplemented medium were sub-cultured onto non-supplemented medium (not shown), indicating that Cr-dependent nonsense suppression did not stem from a prion switch or other heritable change. Translational read-through due to Cr, but not paromomycin, was abolished under anaerobic conditions; this indicates an oxidative basis for Cr-induced mistranslation (Figure 4c).

**Figure 5:**
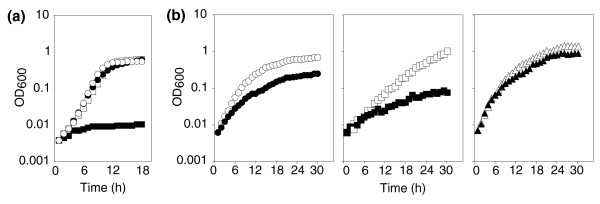
mRNA mistranslation causes chromium toxicity. Exponential phase cells were sub-cultured in 300 μl volumes of YEPD in 48-well plates, and growth (OD_600_) was subsequently monitored at 30°C with continuous shaking in a plate reader. **(a) **Growth of *S. cerevisiae *BY4743 in unsupplemented medium (control; open circles), or in medium supplemented with 0.1 mM Cr (filled circles), or 100 μg ml^-1 ^of the ribosome-targeting drug paromomycin (open squares), or 0.1 mM Cr + 100 μg ml^-1 ^paromomycin (filled squares). **(b) **Growth of *S. cerevisiae *L1494 (wild type; circles), L1583 (error-prone translation; squares) and L1597 (high translational fidelity; triangles) strains in the absence (open symbols) or presence (filled symbols) of 0.1 mM CrO_3_. Typical results from one of three independent experiments are shown.

The hypothesis that induction of mRNA mistranslation causes Cr toxicity was tested first by assaying for synergistic toxicity between Cr and paromomycin. These agents together caused a far stronger growth-inhibitory effect than their combined individual effects (Figure 5a), indicating that paromomycin (which provokes mistranslation via ribosome binding) and Cr target a common process. No synergy was found between H_2_O_2 _and paromomycin (data not shown). Second, we examined Cr resistance in 18S ribosomal RNA mutants that carry out mRNA translation with differing degrees of accuracy [36]. The L1583 mutant, which is characterized by highly error-prone translation, was markedly sensitized to Cr in comparison to the wild type (L1494); in contrast, increased translational accuracy (strain L1597) caused increased Cr resistance (Figure 5b). These results substantiated the proposal that induction of mRNA mistranslation is the main cause of chromium's toxic effect on yeast cells.

## Discussion

The lack of understanding of the cellular and molecular mechanisms that cause metal toxicity has contrasted starkly with our appreciation of the detrimental consequences of metal toxicology for human and animal health. Chromium exposure, for example, is linked with carcinogenicity, liver and kidney necrosis, and allergenicity [8]. In this study, the complete collection of heterozygous deletion mutants of protein-encoding genes in the yeast *S. cerevisiae *was used to determine the contribution of every gene, essential and non-essential, to cellular resistance to chromium. This provides the most comprehensive dataset yet available for elucidating this metal's mode of action. Moreover, we have validated this potential through the novel finding that the induction of mistranslation is a major cause of Cr toxicity. This finding stemmed from an observation that proteasomal functions were over-represented in the annotations of genes that displayed haploinsufficiency in the presence of Cr. Given that most of the proteasomal genes are essential, this result would have been missed in a conventional homozygous-mutant screen, underscoring the importance of including essential gene functions in this type of investigation.

Although metals are not necessarily expected to have essential proteins as their targets [5], we did identify candidate toxicity targets of that type, that is, haploinsufficient essential genes. However, the concept of loss-of-function of an essential protein target (the focus of the drug-induced haploinsufficiency studies [14,15,21,24]) could be less relevant to mode-of-action than toxic gain-of-function, for example, resulting from Cr-induced formation of toxic protein aggregates. In this scenario, candidate protein targets of Cr-mediated toxicity would be among the haploproficient genes. The association between protein aggregation and Cr toxicity remains to be resolved in full. However, we demonstrated that mRNA mistranslation is a primary cause of Cr toxicity, and propose that this toxicity is mediated by aggregation of the mistranslated polypeptides.

It is known that protein (but not DNA) oxidation is required for the process of Cr toxicity [32]. Chromium promotes the generation of superoxide radicals in cells and there are overlaps in the phenotypic effects of Cr and superoxide [32,37,38]. The superoxide-generating provitamin menadione is the only classical pro-oxidant for which haploinsufficiency data are already available [21]. The two genes giving the strongest haploinsufficiency in that report, *GIM1 *and *RPN10*, have functions related to the same principal GO categories identified here for Cr-induced haploinsufficiency: actin and proteasome. Therefore, the present data support the superoxide-related mode of toxicity suggested elsewhere for Cr [32]. These conclusions may be particularly relevant to toxicity in humans as 80% of non-essential gene functions that influence yeast resistance to the superoxide-generating toxicant, paraquat, have highly conserved human homologues [39].

Oxidative stress in yeast is associated with a Gcn2p-dependent repression of translational initiation [40] and similar responses occur in mammalian cells [41]. Combined with translational inhibition additionally at a post-initiation step, this results in a slowdown of protein synthesis that is thought to preclude the potentially deleterious effects of continued mRNA translation under the error-prone conditions of oxidative stress [40]. This strategy of decreased mRNA translation during oxidative stress appears to work in the case of H_2_O_2_, as our data provided no evidence for H_2_O_2_-induced mistranslation. In contrast, the key role for oxygen-dependent mRNA mistranslation in Cr toxicity, revealed here, indicated that translational shutdown is ineffective for Cr. This is despite the fact that assays of translation initiation (C Mascarenhas and CM Grant, personal communication) and [^35^S]methionine incorporation (S Holland and SV Avery, unpublished data) have indicated that Cr provokes a decrease in protein synthesis that is at least as marked as that provoked by H_2_O_2_. Therefore, the ability to respond by decreasing the rate of protein synthesis is not the only factor determining resistance of cells to stressor-induced mistranslation. The specific targeting of the translation process by Cr, indicated by our work, provides a useful new tool for elucidating the molecular mechanisms by which translational fidelity in cells can fail.

## Conclusion

This study has validated the use of the heterozygous yeast mutant collection for mode-of-action discovery beyond therapeutic compounds, with a natural agent not necessarily expected to have essential proteins as its targets. This was also the first study of this nature to exploit the stringency of continuous culture in performing the necessary competitions in a manner that is both highly reproducible and highly sensitive. It is also unique in revealing haploproficient, as well as haploinsufficient, phenotypes with a toxic agent. The screening data presented here provide the research community with an authoritative resource for elucidating the molecular basis of Cr toxicity. Moreover, the data led us to the discovery that Cr induces the mistranslation of mRNA (and increased protein aggregation) and that this is a primary cause of Cr toxicity. Development of new therapies for metal toxicity relies, at least in part, on such advances and these aims should now be closer at hand.

## Materials and methods

### Strains, oligonucleotides and plasmids

The heterozygous deletion strains, in the diploid BY4743 background (*MAT***a**/*MATαhis3Δ *1/*his3Δ *1 *leu2Δ *0/*leu2Δ *0 *met15Δ *0/*MET15 LYS2*/lys2Δ *0 ura3Δ *0/*ura3Δ *0) were obtained from the *Saccharomyces *deletion consortium [42]. The strains were pooled as described elsewhere [22]. BY4743 was used for aggregate extraction, protein labeling and the luciferase assay. The ribosomal mutant strains (L1494, L1597 and L1583) were kindly provided by Dr Susan Liebman (University of Illinois at Chicago).

### Growth conditions

Competition experiments in chemostat culture were carried out according to Colson *et al*. [43] using a small-scale multiple fermenter system (Fedbatch-pro, Das Gip Technology, Julic, Germany). Inoculation with the heterozygote pool and culture in carbon-limited medium were as described by Delneri *et al*. [22], but with the inclusion of CrO_3 _(0.1 mM) or no stressor. In brief, an aliquot (1 × 10^7 ^cells) of the pool of heterozygous strains was inoculated into 120 ml of carbon-limiting medium [44]. These were grown in batch for 24 h at 30°C with shaking at 170 rev min^-1^, before continuous culture was initiated at a dilution rate of 0.1 h^-1 ^and a constant pH of 4.5. Each competition experiment was conducted in two biological replicates for at least 24 generations. Other experiments were with strains cultured individually in YEPD or YNB media [45,46]. Where specified, organisms were cultured in 300 μl volumes in 48-well plates (Greiner Bio-One, Stonehouse, Gloucestershire, UK) with shaking at 30°C in a BioTek Powerwave microplate reader (BioTek, Vinooski, VT, USA). Where specified, an anaerobic atmosphere (H_2 _+ CO_2_) was generated with an Oxoid Gas Generating Kit (Oxoid, Basingstoke, Hampshire, UK).

### Genomic DNA extraction, tag amplification, and hybridization to tag-3 DNA microarrays

Samples (15 ml) of the organisms from competition experiments were collected from the culture outflow as soon as the continuous cultures reached steady state (time zero sample; approximately 72 h after original inoculation) and also after at least 24 generations of steady-state growth. Genomic DNA was extracted from these using the DNA tissue kit (Qiagen, Crawley, West Sussex, UK). The concentration of DNA in the extract was determined using a Nanodrop device (Agilent Technologies, South Queensferry, West Lothian, UK). The universal primers used for amplification of the unique barcodes in the genomic DNA of the heterozygotes and the hybridization protocol are those used by Winzeler *et al*. [10]. Amplifications and hybridizations for each genomic DNA sample were carried out in duplicate, and each sample was from one of two biological replicates of the relevant competition.

### Data analysis

Data from hybridizations were globally normalized by median centering the intensity values from tags corresponding to each heterozygous deletant (two tags per mutant). Log.-ratios for each strain were then calculated between the initial and final chemostat time points [22]. This served to eliminate tag-specific biases and further normalized the data. The log.-ratios were expressed as change (in relative strain abundance) per number of cell generations; the latter correction accounted for differences in the generations elapsed between control and Cr-treated cultures (31 and 24 generations respectively). For each strain, the differences in the mean log.-ratios between the control incubations and incubations with Cr indicated the size of the growth effect of Cr. These growth effects were assessed for significance using the *p *value obtained from an independent-samples *t*-test. To account for multiple testing, false discovery rates (*q*-values) were estimated [47], using the Qvalue v1.0 library implemented in the statistical package R, version 2.4.1. Differences yielding a *q*-value < 0.05 were considered as statistically significant and the corresponding open reading frames selected for further analysis with GoMiner.

Intensity values from tags that do not correspond to deletion mutants were taken as being representative of the background intensity. This was used as a baseline, enabling determination of the presence or absence of individual deletion strains in the experiments. Several strains giving a median signal that was not significantly different to this background were strains that characteristically yield poor hybridization signals [48] (termed 'PH'; Additional data file 1). Data for these were removed from subsequent analyses. Some other strains were lost (out-competed) during competitive culture. Such strains that were not detected in the Cr condition but were in the control condition (termed 'absent'; Additional data file 1) were considered haploinsufficient. Strains that were not detected in the control condition but were in the Cr condition (termed 'present') were considered haploproficient. These strains were included with the relevant haploinsufficient or haploproficient datasets, although the absence of a hybridization signal under either the stressed or the control condition precluded assignment of a *q*-value.

### Protein extraction and metabolic labeling

Protein extraction (total, and the aggregated fraction) was as described in Rand and Grant [49], with the modification of an additional final wash in lysis buffer (minus Igepal), prior to protein quantification with the Bradford assay (Bio-Rad Laboratories, Hemel Hempstead, Hertfordshire, UK). The technique for isolation of aggregates involves solubilization and separation of membrane proteins, so reducing the background of insoluble proteins in aggregate fractions. The term 'aggregated protein', as used in this paper, refers to those fractions that include residual insoluble protein, separated from total protein [49]. For radio-labeling, exponential-phase cells (OD_600 _~0.5) in 25 ml YNB medium were incubated with 1 μl (10 μCi) [^35^S]methionine (MP Biomedicals, Cambridge, Cambridgeshire, UK) ± CrO_3 _for 1 h at 30°C with shaking. Cells were washed twice in chase medium (YNB plus 1 mg ml^-1 ^unlabeled methionine) and protein was extracted and quantified, as above, either immediately or after 1 h incubation in chase medium + CrO_3_. Incorporated isotope was quantified in 5 ml scintillation fluid (Emulsifier Safe, Perkin Elmer, Beaconsfield, Buckinghamshire, UK) using a Packard Tri-Carb 2100TR liquid scintillation analyzer. Incorporation of [^35^S]methionine was expressed as counts-per-minute (cpm) per μg protein.

### Dual luciferase assay

Cultures (5 ml) of cells transformed with the dual-luciferase plasmid [50] (a kind gift from Dr David Bedwell, University of Alabama), were grown to OD_600 _~0.5 and treated with Cu(NO_3_)_2_, CrO_3 _or paromomycin as specified. At intervals, 5 ml of cells were pelleted by centrifugation and resuspended in 60 μl of Passive Lysis Buffer (Promega, Southampton, Hampshire, UK) before vortexing with 40 μl glass beads (0.5 mm diameter, Biospec Products, Bartlesville, OK, USA)) for 10 × 30 s, with a 30 s incubation on ice between each disruption. The subsequent assay was with the Dual Luciferase Assay system (Promega). Extracts were centrifuged at 15,000 *g*, 30 s and 5 μl of supernatant added to 20 μl Luciferase Assay Reagent II. Samples were read in a Berthold Lumat LB9507 luminometer for 10 s, 20 μl of Stop and Glo reagent was added and the luminescence was again read for 10 s. Background measurements obtained for cells that lacked the plasmid were subtracted from test measurements. The derived ratio of luminescence attributable to the firefly versus *Renilla *luciferases indicated the level of UAA mis-translation.

## Abbreviations

GO, Gene Ontology.

## Authors' contributions

SVA and SGO conceived the study. DD and EL performed the genome-wide screen. SH and TS performed all other experiments. IC, KG, SH, DCH, and TR analyzed the data from the screens. SVA, SH and SGO wrote the paper. All authors approved the final manuscript.

## Additional data files

The following additional data are available with the online version of this paper. Additional data file 1 is an Excel workbook that gives the growth data for each heterozygote strain under Cr stress. Additional data file 2 is an Excel workbook that lists the over-represented GO terms among genes that gave significant haploinsufficiency or haploproficiency. Additional data file 3 is a figure showing confirmation of chromium sensitivity in individual heterozygous proteasome mutants. Additional data files 4 and 5 are Excel workbooks that give the raw Affymetrix data and log.-ratios, respectively, for each strain under the control and Cr conditions.

## Supplementary Material

Additional data file 1 Growth data for each heterozygote strain under Cr stress. Click here for file

Additional data file 2 Over-represented GO terms among genes that gave significant haploinsufficiency or haploproficiency. Click here for file

Additional data file 3 Exponential-phase cells were sub-cultured, in 300 μl volumes of YNB in 48-well plates, and growth (OD_600_) was subsequently monitored at 30°C with continuous shaking in a plate reader. Doubling times were determined during the period of exponential growth in the absence or presence of 0.1 mM CrO_3_, and the percent increase in doubling time attributable to Cr (a measure of Cr sensitivity) was calculated for each strain. The values are the means of three independent experiments ± standard error of the mean. Click here for file

Additional data file 4 Affymetrix data for each heterozygote strain under the control and Cr conditions Click here for file

Additional data file 5 Log.-ratio data for each heterozygote strain under the control and Cr conditions Click here for file
